# Interferon-**α** receptor antisense oligonucleotides reduce neuroinflammation and neuropathology in a mouse model of cerebral interferonopathy

**DOI:** 10.1172/JCI169562

**Published:** 2024-02-15

**Authors:** Barney Viengkhou, Christine Hong, Curt Mazur, Sagar Damle, Nicholas B. Gallo, Terry C. Fang, Kate Henry, Iain L. Campbell, Fredrik Kamme, Markus J. Hofer

**Affiliations:** 1 School of Life and Environmental Sciences and the Charles Perkins Centre, The University of Sydney, Sydney, New South Wales, Australia.; 2 Ionis Pharmaceuticals, Carlsbad, California, USA.; 3 Biogen Inc, Cambridge, Massachusetts, USA.

**Keywords:** Inflammation, Neuroscience, Drug therapy, Innate immunity, Neurological disorders

## Abstract

Chronic and elevated levels of the antiviral cytokine IFN-α in the brain are neurotoxic. This is best observed in patients with genetic cerebral interferonopathies such as Aicardi-Goutières syndrome. Cerebral interferonopathies typically manifest in early childhood and lead to debilitating disease and premature death. There is no cure for these diseases with existing treatments largely aimed at managing symptoms. Thus, an effective therapeutic strategy is urgently needed. Here, we investigated the effect of antisense oligonucleotides targeting the murine IFN-α receptor (*Ifnar1* ASOs) in a transgenic mouse model of cerebral interferonopathy. Intracerebroventricular injection of *Ifnar1* ASOs into transgenic mice with brain-targeted chronic IFN-α production resulted in a blunted cerebral interferon signature, reduced neuroinflammation, restoration of blood-brain barrier integrity, absence of tissue destruction, and lessened neuronal damage. Remarkably, *Ifnar1* ASO treatment was also effective when given after the onset of neuropathological changes, as it reversed such disease-related features. We conclude that ASOs targeting the IFN-α receptor halt and reverse progression of IFN-α–mediated neuroinflammation and neurotoxicity, opening what we believe to be a new and promising approach for the treatment of patients with cerebral interferonopathies.

## Abstract 2

<p>Antisense oligonucleotides targeting the interferon alpha receptor efficiently prevent and reducing disease in a mouse model of cerebral interferonopathy.</p>

## Graphical abstract

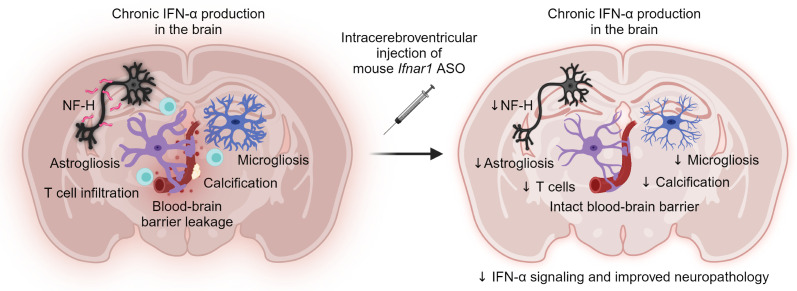

## Introduction

Despite the important role type I IFNs like IFN-α play in the host antiviral immune response, its actions in the central nervous system (CNS) can be debilitating. This is particularly evident in patients with cerebral interferonopathies — a group of diseases characterized by chronically elevated IFN-α levels in the CNS (1–4). Cerebral interferonopathies are heterogenous and arise from heritable and de novo genetic mutations, autoimmunity, chronic viral encephalitis, or have no known etiology (1–4). The prototypical example is the genetic disorder Aicardi-Goutières syndrome (AGS). AGS is an early onset and progressive childhood disease with brain pathology in patients characterized by reactive astrocytes and microglia, vasculopathy, leukocyte infiltration, perivascular calcification, white matter abnormalities, and progressive neurodegeneration (3, 5–9). Clinically, patients can display irritability and/or abnormal movements that develop into ataxia or seizures and have reduced life expectancy (3, 5–9). In the CNS of patients with AGS, excessive IFN-α is produced by astrocytes, among other cell types (6, 10, 11), and is at higher levels in the cerebral spinal fluid than blood (12, 13). Together, these pathological features have led to the classification of AGS as a leukodystrophy and astrocytopathy (14). Importantly, there are currently no effective therapies for cerebral interferonopathies and personalized treatment plans are primarily aimed at managing symptoms (15).

The development of effective treatments is hindered by the largely unknown cellular and molecular mechanisms involved in driving the pathology of AGS and related cerebral interferonopathies. Currently, two main strategies are being pursued; targeting either (a) the processes leading to increased IFN-α production or (b) the signaling pathway activated by IFN-α after receptor binding (15, 16). Of note, targeting the molecular processes leading to IFN-α production may be problematic given the genetic diversity underlying AGS, which is caused by mutations in at least 9 genes (17, 18). By contrast, targeting the receptor for type-I–IFN signaling should be a more promising strategy as it would abrogate the biological actions of IFN-α independent of disease etiology. Activation of the signaling pathway by IFN-α begins with its binding to the heterodimeric type I IFN receptor, comprised of IFNAR1 and IFNAR2, which activates Janus kinases (JAKs) that, in turn, induce several phosphorylation cascades in parallel, leading to the regulation of hundreds of IFN-regulated genes (19).

A therapeutic strategy aimed at targeting IFNAR employs target-specific antisense oligonucleotides (ASOs). ASOs have been shown to regulate the expression of target gene transcripts with high specificity (20). The therapeutic efficacy of ASOs is evident in patients with spinal muscular atrophy, where ASO treatment has led to marked overall improvements in physical and motor abilities (21). Accordingly, ASO-based drugs are currently in clinical trials for the treatment of several rare genetic disorders and neurodegenerative diseases (22–24). Here, we investigated the therapeutic effect of dosing murine *Ifnar1*-specific ASOs in an established transgenic mouse model of cerebral interferonopathy.

While animal models based on common genetic mutations of AGS have aided in our understanding of how gene defects result in increased IFN-α signaling, these models have failed to recapitulate the neuropathology and neurological clinical hallmarks observed in patients with AGS (25). By contrast, mice with chronic IFN-α production in the brain driven by GFAP-expressing astrocytes (termed “GIFN” mice) closely mirror the neuropathological and clinical features observed not only in the brain of patients with AGS, but also of patients with other cerebral interferonopathies (26, 27). As such, the GIFN mouse model was used to assess the therapeutic effects of mouse-specific *Ifnar1* ASOs (herein referred to as *Ifnar1* ASOs) in IFN-α–induced cerebral molecular and cellular pathology. Intracerebroventricular injection of mouse *Ifnar1* ASOs halted the progression of neuropathology and partially reversed the cerebral transcriptomic signature in GIFN mice. Remarkably, treatment of older GIFN mice with established CNS pathology was still effective at halting and even reversing some neuropathological features of the disease. We believe that these results demonstrate that ASOs targeting *IFNAR1* represent a promising new tool to treat AGS and related cerebral interferonopathies.

## Results

### Improved neuropathology in GIFN mice treated with mouse Ifnar1 ASOs.

**Figure 1:**
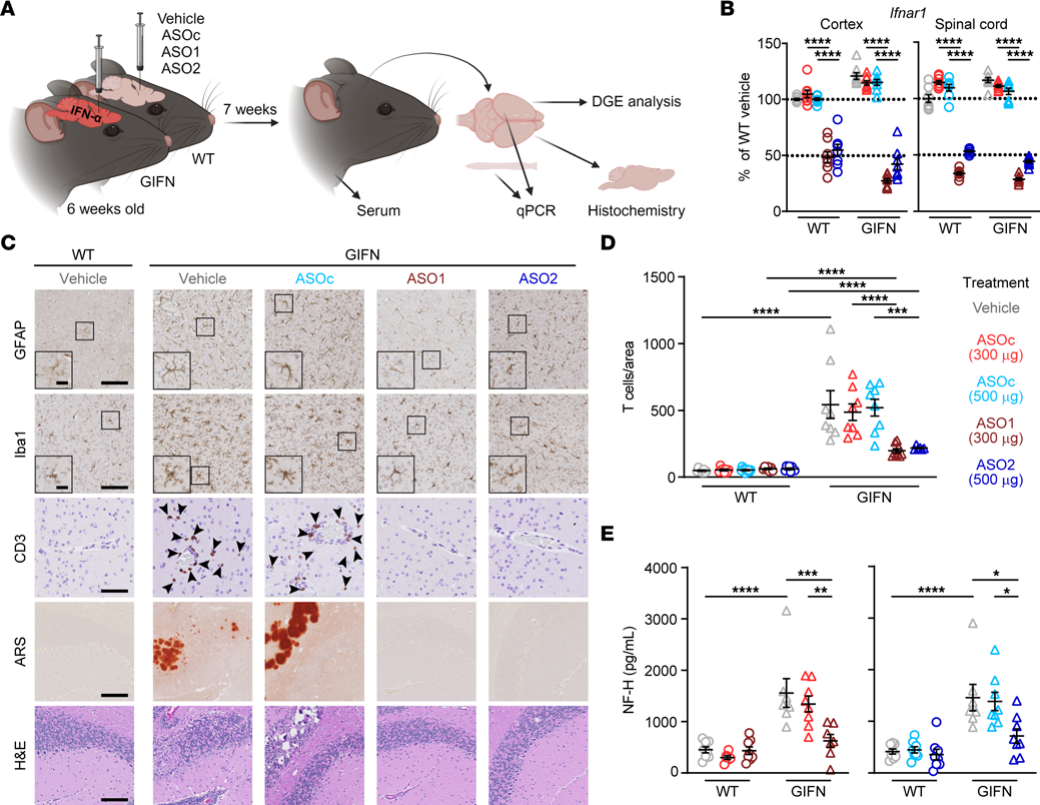
ASO-mediated knockdown of *Ifnar1* reduces neuropathology in GIFN mice. (**A**) Schematic of experiment. (**B**) Real-time qPCR of relative *Ifnar1* expression in cortex and spinal cord of mice. *n* = 7–8 mice per genotype per treatment. Mean and SEM shown. (**C**) Representative images of GFAP, Iba1, and CD3 IHC in the cortex and alizarin red S (ARS) and H&E staining in the cerebellum of vehicle and treated WT and GIFN mice. Scale bars: 100 μm and 20 μm in inserts. Arrowheads indicate CD3^+^ cells. (**D**) Quantification of the number of CD3^+^ T cells in brain sections normalized to area (*n* = 6–10 mice per genotype per treatment). (**E**) Relative level of serum neurofilament heavy subunit (NF-H) in treated WT and GIFN mice (*n* = 6–10 mice per genotype per treatment). Each point is a mouse. Mean and SEM shown. *P < 0.05, **P < 0.01, ***P < 0.001, and ****P < 0.0001, by 2-way ANOVA with Tukey’s post test.

Delivery of ASOs into the cerebrospinal fluid leads to widespread distribution in the CNS (28) and bypasses the blood-brain barrier, enabling direct ASO activity in brain cells. To test the efficacy of *Ifnar1* ASOs in treating cerebral interferonopathies, WT and GIFN mice at 6 weeks of age were given a bolus intracerebroventricular dose of vehicle, control ASO (ASO_C_), or mouse *Ifnar1* ASOs (ASO1 or ASO2) and changes were analyzed 7 weeks later (Figure 1A). Young mice were used for this experiment before the development of pronounced neuropathological changes (29). Real-time quantitative PCR (qPCR) showed 45%–72% knockdown of *Ifnar1* mRNA in the cortex and spinal cord across the 2 *Ifnar1* ASOs and genotypes compared with vehicle and ASO_C_ treatments (Figure 1B). This demonstrates that a localized injection of *Ifnar1* ASOs results in a widespread reduction of *Ifnar1* transcripts within the CNS.

Next, IHC was performed to screen for changes in neuropathological features in *Ifnar1* ASO-dosed GIFN mice (Figure 1C). There were no observable gross differences or pathological changes in the brains of WT mice receiving any of the treatments. By contrast, brains from GIFN mice in the vehicle or ASO_C_ control groups displayed gliosis. GFAP-positive astrocytes were hypertrophic with fewer and less complex processes, while Iba1-positive microglia had thicker cell bodies with complex processes that at times wrapped around calcified deposits, confirming our previous observations in these mice (30). An influx of T cells has been described in the GIFN model (26) and represents a possible mechanism of inflammation-driven neuropathology (31). Accordingly, there was increased infiltration of CD3^+^ T cells (Figure 1, C and D) as well as the presence of calcification and tissue destruction in the brains of vehicle and ASO_C_-treated GIFN mice. By contrast, treatment of GIFN mice with either *Ifnar1* ASO1 or ASO2 resulted in reduced gliosis, based on an intermediate morphology displayed by astrocytes and microglia and absence of calcification or tissue destruction. Notably, *Ifnar1* ASO treatment led to a robust reduction in T cell recruitment by a factor of 2.4–2.5 fold compared with vehicle and ASO_C_-treated GIFN mice.

As an indicator of neuropathological improvement with *Ifnar1* ASO treatment, serum levels of the neuronal damage marker neurofilament heavy subunit (NF-H) (32) were determined. Compared with vehicle-treated WT mice, GIFN mice had a significant 3.5-fold increase in serum NF-H levels (Figure 1E). Treatment with ASO_C_ had no significant effect on NF-H levels, while *Ifnar1* ASO1 and ASO2 treatment of GIFN mice resulted in a significant 2-fold reduction in serum NF-H levels, compared with ASO_C_-treated GIFN mice. To investigate neuronal damage, we quantified levels of apoptotic neurons in the cerebellum and cortex. There was a significant increase in the number of apoptotic neurons and nonneuronal cells in the cerebellum of GIFN mice compared with WT mice, which were diffusely distributed throughout the cerebellum (Supplemental Figure 1; supplemental material available online with this article; https://doi.org/10.1172/JCI169562DS1). *Ifnar1* ASO-treatment significantly reduced the number of apoptotic cells in transgenic mice to levels similar to those seen in WT mice. In the cortex, there were no detectable changes in the number of apoptotic cells between WT and GIFN mice (Supplemental Figure 1C), probably the consequence of lower transgene expression in this area compared with the cerebellum. We also looked at the neurogenic niche of the subventricular zone (SVZ) by staining with PCNA, a proliferation marker. There was no difference in the number of PCNA-positive neuronal progenitor cells in the subventricular zone of WT and GIFN mice independent of treatment (Supplemental Figure 2, A and B). However, there was a significant increase in the number of PCNA-positive cells in the cerebellum of vehicle or ASO_C_-treated GIFN mice compared with WT mice, and this number was reduced in *Ifnar1* ASO-treated GIFN mice to levels similar to those seen in WT mice (Supplemental Figure 2, A and C). White matter loss can occur with neuronal loss, and, therefore, we quantified myelin basic protein (MBP) by IHC. There was no difference in staining pattern across genotypes or treatments, indicating there was no detectable loss of myelin (Supplemental Figure 3). This suggests that *Ifnar1* ASO treatment limits neuronal damage in the brains of GIFN mice, mirroring the neuropathological findings. Together, these results demonstrate the marked positive impact a single dose of *Ifnar1* ASO has on improving the cerebral pathology of GIFN mice.

### Transcriptomic changes reflect the reduced neuropathology in Ifnar1 ASO-treated GIFN mice.

**Figure 2:**
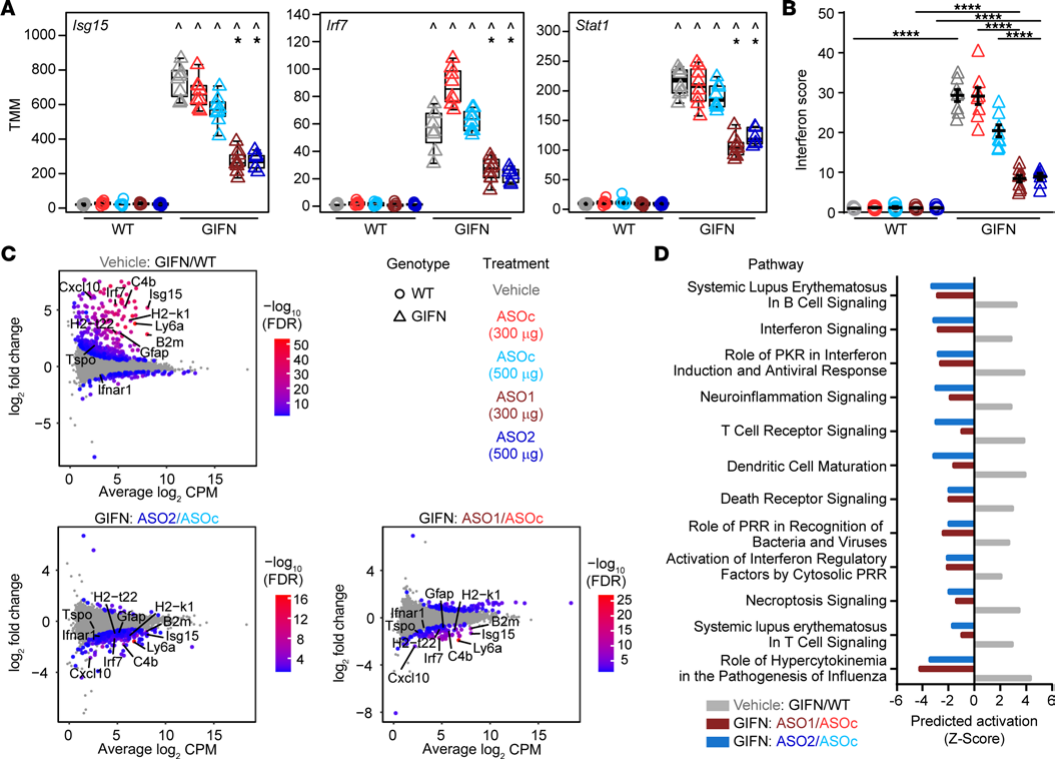
Transcriptomic changes with ASO treatment in GIFN mice reflect improved neuropathology. (**A**) Box plots of normalized expression levels (weighted trimmed mean of M values, TMM) of IFN-stimulated genes. Points are individual mice. ^, compared with corresponding treatment in WT mice; *, compared with corresponding ASO_c_ treatment, indicating *P* < 0.05 by quasi-likelihood F-test. (**B**) IFN score (*n* = 6–9 mice per genotype per treatment; each point is a mouse and mean and SEM are shown. ****P < 0.0001, by 2-way ANOVA with Tukey’s post test). (**C**) Mean-difference plots with key genes indicated that are associated with pathways in **D**. Gray dots represent genes that are not significantly regulated. (**D**) Predicted activation status of significantly enriched pathways identified by Ingenuity Pathway Analysis of all significantly regulated genes for each comparison.

In addition to the neuropathological analyses, we performed DGE-Seq on brain cortex samples to determine the impact of ASOs on the transcriptome. We examined the regulation of key genes associated with type-I–IFN signaling (Figure 2A). There was a robust reduction of *Isg15*, *Irf7,* and *Stat1* expression levels in GIFN mice treated with either *Ifnar1* ASO compared with vehicle and ASO_C_-treated GIFN mice. We next calculated the IFN score, a biomarker used in clinical practice to assess enhanced type-I–IFN signaling in patients with AGS (8). The IFN score was significantly higher in vehicle-treated GIFN mice compared with WT mice, and treatment of GIFN mice with *Ifnar1* ASOs resulted in a significant decrease of the IFN score (Figure 2B). To further investigate the CNS effects of *Ifnar1* knockdown, differential gene analysis was performed. Mean-difference plots revealed a large number of genes that were significantly upregulated in vehicle-treated GIFN mice compared with WT mice (Figure 2C). Conversely, many genes were downregulated in *Ifnar1* ASO1 or ASO2-treated GIFN mice compared with ASO_C_-treated GIFN mice, as visualized using a heatmap (Supplemental Figure 4A). These analyses also revealed reduced expression of many genes principally upregulated in vehicle-treated GIFN mice compared with *Ifnar1* ASO1 or ASO2-treated GIFN mice. Functional analysis was next performed to assess the potential physiological impact of the changes in gene expression. Since *Ifnar1* ASO treatment of GIFN mice leads to the reduced expression of a large number of genes, we used barcode plots to visualize how changes in gene expression were associated with ontology terms (Supplemental Figure 4B). Compared with vehicle-treated WT mice, vehicle-treated GIFN mice expressed a higher number of genes associated with innate immune response and antigen processing and presentation. Importantly, relative to ASO_C_, dosing of GIFN mice with *Ifnar1* ASO1 or ASO2 decreased expression of genes associated with both processes. Given the reversal in enrichment of these terms, we predicted the activation status of several cellular pathways (Figure 2D). There was increased activation of pathways associated with interferon signaling, neuroinflammation signaling, T cell receptor signaling, death receptor signaling, and role of hypercytokinemia in the pathogenesis of influenza in vehicle-treated GIFN mice compared with vehicle-treated WT mice. Many of these terms reflect the changes in neuropathology in vehicle-treated GIFN mice. Importantly, these pathways were predicted to have reduced activation with *Ifnar1* ASO-treatment in GIFN mice compared with ASO_C_-treated GIFN mice. Thus, *Ifnar1* ASOs effectively dampen and prevent the IFN-α–induced hyperinflammatory transcriptomic landscape in the brains of GIFN mice, aligning with the improvement in neuropathology observed.

### Chronic neuropathology in GIFN mice is reversed following delayed treatment with Ifnar1 ASOs.

**Figure 3:**
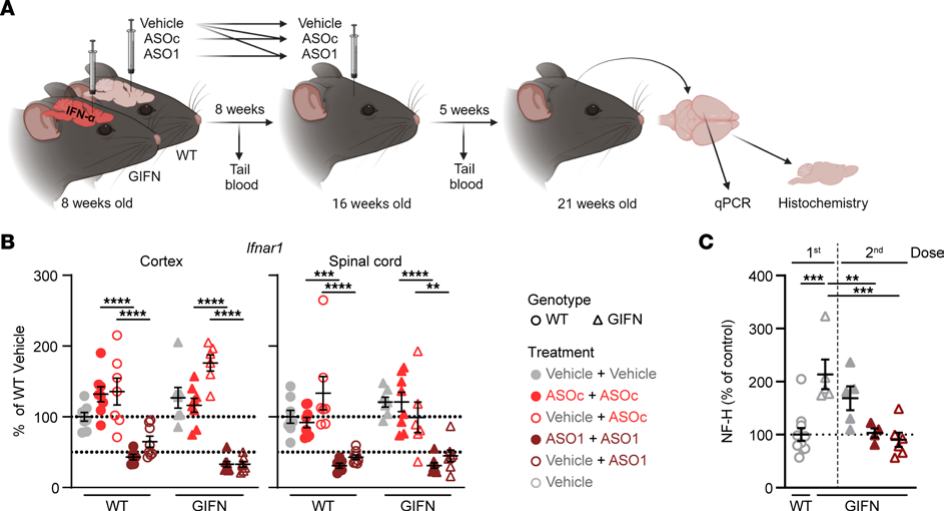
Delayed *Ifnar1* ASO dosing reduces *Ifnar1* transcript and NF-H disease biomarker levels in GIFN mice with established neuropathology. (**A**) Schematic of the experiment. Tail blood was taken 1 week prior to the second dose and/or 1 week prior to euthanasia. (**B**) Real-time qPCR of relative *Ifnar1* expression in cortex and spinal cord of mice (*n* = 6–8 per genotype per treatment). Each point is a mouse and mean and SEM shown. 2-way ANOVA with Tukey’s post test. (**C**) Relative level of serum NF-H in treated WT and GIFN mice (*n* = 5–6 mice per genotype per treatment). Each point is a mouse and mean and SEM are shown. **P < 0.01, ***P < 0.001, and ****P < 0.0001, by 1-way ANOVA with Tukey’s post test.

The above data show that *Ifnar1* ASOs halt the neuropathological progression of disease in GIFN mice by dampening the inflammatory environment. As the neuropathological features of GIFN mice are progressive and worsen with age (26, 27, 29), we next explored whether *Ifnar1* ASOs maintain their positive effect over time and/or whether they can reverse established neuropathology. We hence tested repeat treatment with *Ifnar1* ASOs at 8 and 16 weeks of age as well as delayed treatment of GIFN mice at 16 weeks of age when neuropathology was already present. Treated mice were subsequently euthanized at 21 weeks of age (Figure 3A). Specifically, the treatments for the repeat dosing groups were vehicle + vehicle, ASO_C_ + ASO_C_, and ASO1 + ASO1, and for the delayed groups, vehicle + ASO_C_ and vehicle + ASO1. Additionally, some vehicle-treated mice were euthanized at 16 weeks of age to assess neuropathological changes at the time of the second dose.

Following repeat dosing with *Ifnar1* ASO1, real-time qPCR demonstrated that *Ifnar1* mRNA was maintained below 50% of WT levels (Figure 3B). This was similar for the delayed dose in GIFN mice. Further, knockdown of *Ifnar1* mRNA did not differ significantly between repeat (ASO1+ASO1) dosing or a delayed dose (vehicle + ASO1) of *Ifnar1* ASO.

In GIFN mice, serum NF-H was elevated both after the first and second dose of vehicle compared to WT mice (Figure 3C). By contrast, there was a significant reduction in NF-H levels in GIFN mice that received repeat doses (ASO1 + ASO1) or the delayed dose (vehicle+ASO1) of *Ifnar1* ASO. In addition, there were fewer apoptotic neurons and nonneuronal cells in the cerebellum of GIFN mice that had received repeat (ASO1 + ASO1) or delayed (vehicle + ASO1) ASO doses compared with vehicle + vehicle–treated GIFN mice (Supplemental Figure 5, A and B). Small clusters of apoptotic cells were observed in areas of the cerebellum associated with tissue destruction. There were no detectable differences in the number of apoptotic cells in the cortex (Supplemental Figure 5C). Further, there were no changes in the number of proliferating cells in the SVZ but there was significant reduction of PCNA-positive cells in the cerebellum of GIFN mice with repeat (ASO1 + ASO1) and delayed (vehicle + ASO1) doses compared with vehicle + vehicle–treated GIFN mice (Supplemental Figure 6). Together, these results indicate that *Ifnar1* ASO successfully reduced ongoing neuronal damage over an extended period of time and was also able to reduce neuronal damage when given after disease onset.

**Figure 4:**
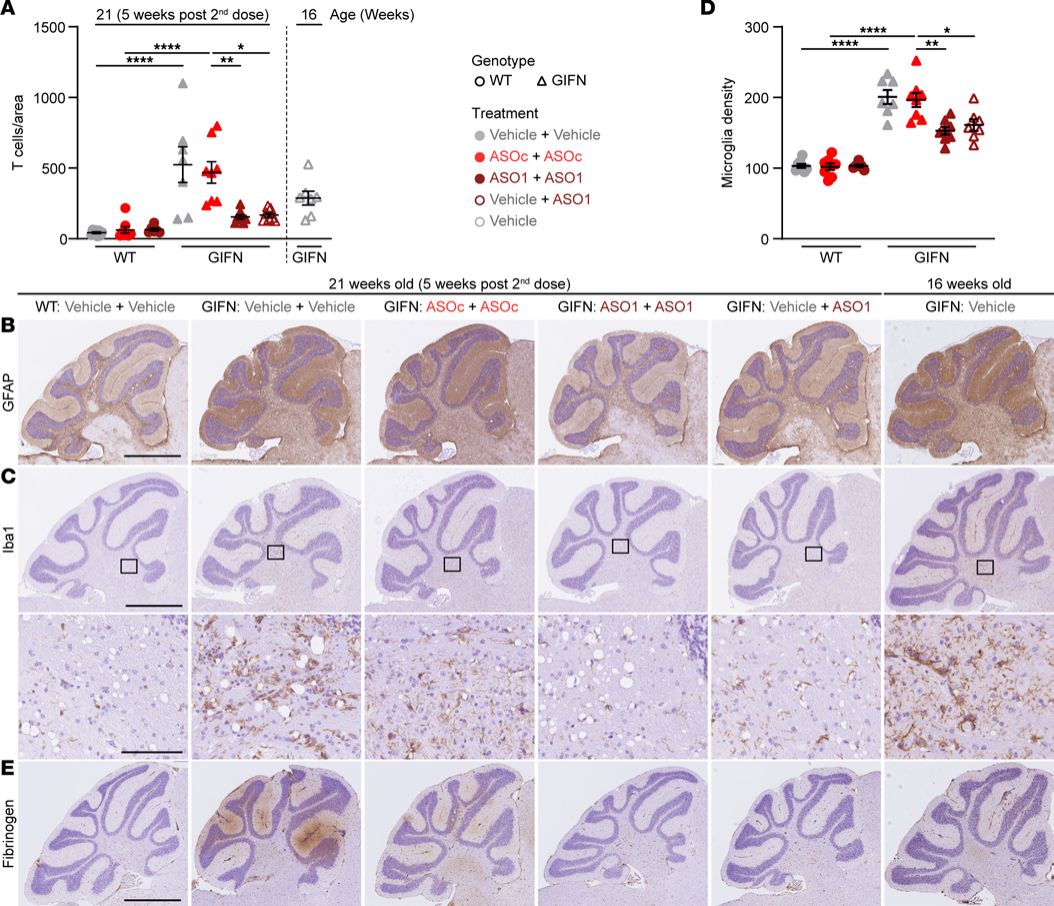
Delayed dosing of GIFN mice with *Ifnar1* ASO reverses neuropathology. (**A**) Quantification of CD3^+^ T cells in brain sections of 21-week-old (5 weeks after second dose) WT or GIFN mice treated with vehicle + vehicle, ASO_c_ + ASO_c_, *Ifnar1* ASO1 + *Ifnar1* ASO1, or vehicle + *Ifnar1* ASO1. Counts were normalized to area. Representative images of (**B**) GFAP and (**C**) Iba1 IHC in treated WT and GIFN mice with (**D**) quantification of microglia per mm^2^ and (**E**) fibrinogen IHC to indicate blood-brain barrier leakage. Scale bar: 1 mm and 100 μm for high magnification. (**A** and **D**) Each point is a mouse and mean and SEM shown. *P < 0.05, **P < 0.01, and ****P < 0.0001, by 1-way ANOVA with Tukey’s post test.

To confirm this, detailed neuropathological analysis was performed. Quantification of immunostaining for CD3 revealed that infiltration of T cells into the brains of GIFN mice was significantly reduced, 3.1-fold, following repeat dosing of *Ifnar1* ASO (ASO1 + ASO1) compared with ASO_C_-treated GIFN mice (ASO_C_ + ASO_C_) (Figure 4A). Similarly, the delayed dose (vehicle + ASO1) in GIFN mice resulted in a 2.8-fold reduction in T cell counts. There was also a nonsignificant reduction in T cell counts in 21-week-old GIFN mice that received the delayed dose (vehicle + ASO1) compared with 16-week-old vehicle-treated GIFN mice, suggesting *Ifnar1* ASO treatment reversed the infiltration of T cells. IHC for GFAP showed increased staining throughout the brain in GIFN mice from the vehicle + vehicle and ASO_C_ + ASO_C_ groups (not shown), with most pronounced signal in the cerebellar molecular layer and in deeper cerebellar nuclei (Figure 4B). Repeated *Ifnar1* ASO dosing (ASO1 + ASO1) of GIFN mice reduced GFAP staining in the cerebellum compared with control-treated GIFN mice (vehicle + vehicle and ASO_C_ + ASO_C_ groups). This was similarly seen for GIFN mice receiving the delayed dose (vehicle + ASO1). Importantly, GFAP staining was stronger in 16-week-old vehicle-treated GIFN mice compared with GIFN mice receiving the delayed dose (vehicle + ASO1), further suggesting that delayed *Ifnar1* ASO treatment reversed the changes in GFAP staining. Similar to GFAP staining, Iba1 staining was markedly increased in GIFN mice in the vehicle + vehicle and ASO_C_ + ASO_C_ groups, compared with WT mice in the vehicle + vehicle group (Figure 4C). In addition to increased staining, there was a significant 2-fold increase in cortical microglia density in control-treated GIFN mice (vehicle + vehicle and ASO_C_ + ASO_C_ groups) compared with WT mice; but repeat (ASO1 + ASO1) and delayed (vehicle + ASO1) dosing of GIFN mice resulted in a significant, 50%, reduction in microglia density (Figure 4D). Overall, microgliosis was reduced in GIFN mice with repeated *Ifnar1* ASO dosing (ASO1 + ASO1) or delayed dosing (vehicle + ASO1). Additionally, there was reduced Iba1 staining in 21-week-old GIFN mice with a delayed dose (vehicle + ASO1) compared with 16-week-old vehicle-treated GIFN mice, showing that delayed *Ifnar1* ASO treatment could reverse reactive microgliosis. Finally, using fibrinogen IHC as an indicator of blood-brain barrier integrity (33), we found increased staining in the cerebellum, both in the molecular layer and deep cerebellar nuclei, in 21-week-old GIFN mice in the vehicle + vehicle and ASO_C_ + ASO_C_ groups compared with WT controls (Figure 4E). By contrast, parenchymal staining for fibrinogen was largely absent in GIFN mice with repeat *Ifnar1* ASO dosing (ASO1 + ASO1), suggesting *Ifnar1* ASO treatment prevented the increase in parenchymal fibrinogen and protects against blood-brain barrier leakage in GIFN mice. There was faint fibrinogen staining in 16-week-old vehicle-treated GIFN mice, which was largely absent in 21-week-old GIFN mice after the delayed dose (vehicle + ASO1). This suggests that blood-brain barrier leakage may be a relatively late phenomenon in GIFN mice and that delayed *Ifnar1* ASO treatment potentially reversed the development of blood-brain barrier leakage.

## Discussion

There is a need to develop effective therapeutic strategies to treat patients with debilitating cerebral interferonopathies. In the current study, we assessed the therapeutic efficacy of targeting type-I–IFN signaling using *Ifnar1* ASOs in the GIFN mouse model of CNS interferonopathy. Importantly, intracerebroventricular injection of mouse *Ifnar1* ASOs in GIFN mice successfully halted and/or rescued neuroinflammatory and neuropathological phenotypes compared with control ASO-treated mice. This included a robust reduction in type-I–IFN signaling, absence of observable tissue destruction, reduced reactivity of microglia and astrocytes, reduced leukocyte infiltration and neuronal apoptosis, and prevention of blood-brain barrier integrity loss. Collectively, we believe that these results demonstrate that *IFNAR1* ASOs are a promising therapeutic approach for patients with cerebral interferonopathies.

Intracerebroventricular mouse *Ifnar1* ASO treatment effectively reduced *Ifnar1* transcript levels in the brain. This occurred throughout the CNS of WT and GIFN mice at similar efficacies despite the hyperinflammatory environment in the brain of GIFN mice. Importantly, only minor changes in some transcript levels were seen with control ASO_C_ treatment in either WT or GIFN mice, suggesting no substantial target-independent effects of ASO treatment, matching results in other studies (34, 35). Similarly, *Ifnar1* mRNA knockdown in WT mice had little effect on the global transcriptome, mirroring the minor transcriptomic differences seen between WT and *Ifnar1*-deficient mice (36). By contrast, in GIFN mice, *Ifnar1* ASO treatment reduced the downstream effects of IFN-α on gene expression and rescued the brain transcriptome of GIFN mice from a hyperinflammatory signature. Thus, *Ifnar1* ASOs are an effective tool to successfully downregulate activation of IFN-α signaling pathways in vivo.

The shift in the transcriptome of GIFN mice due to *Ifnar1* ASO treatment translated to reduced neuropathology. In particular, reactive gliosis, blood-brain barrier leakage, T cell infiltration, cerebral calcification, and neurodegeneration, including apoptotic cell death, were milder or absent in *Ifnar1* ASO-treated GIFN mice. This demonstrates that *Ifnar1* ASO treatment is effective in ameliorating diffuse brain disease caused by excessive type-I–IFN signaling. Importantly, results from delayed *Ifnar1* ASO dosing of GIFN mice, after the development of advanced neuropathology, demonstrates that such treatment remains effective, leading to regression of some aspects of type-I-interferon–induced neuropathology in diseased mice, including gliosis and blood-brain barrier disruption. This is highly relevant in a clinical setting, as patients typically present after the onset of disease and development of brain pathology (37–44). Altogether, these findings confirm that aberrant IFN-α signaling in the CNS is a critical disease mechanism in cerebral interferonopathies and, most importantly, is amenable to therapeutic intervention — even when disease is advanced.

While ASO treatment was successful at limiting excessive IFN-α signaling, it is important to note that the observed improvement of neuropathology in *Ifnar1* ASO-treated GIFN mice occurred in the presence of residual IFN-α signaling, as evidenced by increased IFN scores in *Ifnar1* ASO-treated GIFN mice. This indicates that *Ifnar1* ASO treatment is an effective strategy to titrate IFN-α signaling below the disease-inducing threshold. The existence of such a threshold of IFN-α is in line with the observed positive correlation between IFN-α levels and disease severity in humans with systemic and cerebral interferonopathies (45) and in 2 lines of GIFN mice with low and moderate transgene expression (26, 27, 46).

In GIFN mice, IFN-α is produced chronically by astrocytes, replicating findings in postmortem tissue from patients with AGS and studies using induced pluripotent stem cells derived from patients, that have identified astrocytes as the main source of IFN-α and proinflammatory cytokines (11, 47). Furthermore, ASOs show efficacy against all cell types in the CNS (48). Thus, we expect *IFNAR1* ASOs to reduce type-I–IFN signaling in the CNS of patients with AGS, even if cell types beyond astrocytes are engaged. However, future studies could investigate the contribution of different cell types such as astrocytes or microglia to disease pathogenesis as a basis to develop cell-type specific delivery of ASOs.

In summary, this study demonstrates that *Ifnar1* ASOs significantly reduced many key molecular, cellular and systems disease manifestations in a mouse model of cerebral interferonopathies, offering hope for individuals and families suffering from such conditions like AGS. Beyond what we believe to be a novel ASO strategy, additional treatment modalities, such as JAK inhibitors, are being tested with some success in a subgroup of patients (37–44). Unlike these inhibitors, which require daily or multi-daily oral dosing, commercial ASO-based drugs for CNS indications, such as nusinersen, are delivered intrathecally 3 to 4 times a year due to their prolonged stability and long-lasting efficacy (49, 50). Due to the relatively low dose, infrequent dosing paradigm, and dilution of ASO in large blood volume following clearance from CSF, systemic exposure is minimal (22, 28) and limits ASO-mediated target knockdown predominantly to the CNS. As such, intrathecally dosed ASOs have much higher cerebral exposure than orally dosed JAK inhibitors (38, 43) and a lower risk for side effects of JAK inhibition, such as systemic immunosuppression, cancer, and major adverse cardiovascular events (51–54). Compared with JAK inhibitors, ASOs have a different set of potential safety concerns, such as proinflammatory effects through TLR9 binding (55). These are, however, sequence-dependent effects, and, through screening and development, safe and effective ASO medicines have been developed, as exemplified by nusinersen for spinal muscular atrophy (56) and tofersen for SOD1-dependent ALS (57).

Overall, these results demonstrate that *Ifnar1* ASOs ameliorate and reduce neuroinflammation and neuropathology caused by chronically elevated IFN-α levels in the brain even at an advanced stage of disease. Hence, we believe that *IFNAR1* ASOs are a promising therapeutic strategy to remedy the debilitating impacts of AGS and related cerebral interferonopathies.

## Methods

### Sex as a biological variable.

Our study examined male and female animals, and similar findings are reported for both sexes.

### Mice.

All mice were housed and maintained under specific pathogen-free conditions in the animal house facility in the School of Life and Environmental Sciences (The University of Sydney) or Taconic Biosciences (San Diego, California, USA) or at Ionis Pharmaceuticals (Carlsbad, California, USA), receiving food and water ad libitum. Mice were maintained by inbreeding, and GIFN mice (26, 27) were originally obtained from the Scripps Research Institute (La Jolla, California, USA) where they were developed by I. L. Campbell. Mice were euthanized using isoflurane or CO_2_.

### ASO treatments.

Intracerebroventricular bolus injections in mice were performed as described previously (58) with some modifications. Briefly, ASOs were delivered in a 10 μl volume administered over 1 minute into the right ventricle. Injection coordinates were: 0.3 mm anterior and 1.0 mm to the right of bregma and 3 mm deep. All ASOs used were 5-10-5 MOE gapmers (Ionis Pharmaceuticals). An ASO_C_ was used for each *Ifnar1* ASO, 5′-CCTATAGGACTATCCAGGAA-3′ and 5′-AATGCTTTCATAACTTCCAC-3′, respectively for ASO1 and ASO2. Neither have a substantial sequence match in the mouse transcriptome. *Ifnar1* ASOs were ASO1: 5′-CTGGTATCTTTTCTACATAA-3′, which targets mouse *Ifnar1* RNA in exon 5, and ASO2: 5′-GTTAACATCTTTCTGACTCT-3′, which targets intron 2 in mouse *Ifnar1* RNA.

### RNA extraction and qPCR.

Tissue samples were homogenized in Trizol (Thermo Fisher Scientific) using FastPrep beads (MP Biomedicals) on a Precellys 24 homogenizer (Bertin Technologies). RNA was isolated and DNase treated on an RNeasy 96-well plate (Qiagen). One step real-time qPCR was run in 96-well format on a StepOnePlus PCR instrument (Thermo Fisher Scientific), using Taqman probes (Integrated DNA Technologies) and Express PCR mix (Thermo Fisher Scientific). Ct values were then linearized against a standard curve generated by diluting RNA. Expression levels of *Ifnar1* were normalized to *Ppia* (Cyclophilin A).

### Histology, IHC, and immunofluorescence.

Sagittal brains were fixed in 10% neutral buffered formalin (Sigma-Aldrich) and processed into paraffin (Leica). Sections (5 μm) were dewaxed and rehydrated through an ethanol series. H&E staining was performed at the Histopathology Facility (Department of Pathology, The University of Sydney). To demonstrate calcification, 2% alizarin red S (ARS; Sigma-Aldrich) pH 4.2 was used to stained rehydrated sections before washing in acetone (Chem-Supply) and then coverslipped.

Microglia and astrocytes were labeled with antibodies against Iba1 (1:1,000; 019-19741, Wako) and GFAP (1:1,000; Z0334, Dako), respectively. Antigen retrieval was performed in 25 mM Tris pH 8 for Iba1 and 25 mM Tris pH 9 for GFAP in a steamer for 35 minutes. Sections were blocked with 0.3% H_2_O_2_ and then 1% goat serum in 137 mM NaCl, 2.7 mM KCl, 10 mM Na_2_HPO_4_, 1.8 mM KH_2_PO_4_, 0.1% triton-X, and 0.05% tween-20. Primary antibodies were incubated overnight at 4°C. Secondary antibodies (goat anti-rabbit IgG, BA-1000, Vector Laboratories) were incubated for 30 minutes prior to the application of ABC kit (PK-6100, Vector Laboratories) for 30 minutes. Antigen location was revealed using 3,3′-diaminobenzidine (SK-4100, Vector Laboratories) prior to counterstaining with hematoxylin (MHS16, Sigma-Aldrich) and then coverslipping. Additionally, IHC was performed on a Ventana Discovery Ultra staining system (Roche) with Iba1, GFAP, CD3 (ab5690, Abcam), MBP (ab218011, Abcam), and fibrinogen (ab189490, Abcam). Detection was done using either the ChromoMap DAB kit or the DABmap kit (Ventana).

For immunofluorescence, proliferating cells were labelled with PCNA (1:4,000; A12427, Abclonal) after antigen retrieval with 10 mM citrate buffer. Following blocking with serum, the primary antibody was incubated overnight at 4°C. Antigen location was revealed with anti-rabbit CF-647 (1:500; SAB4600393, Sigma-Aldrich) incubated for 30 minutes with DAPI (1:1,000) before washing, coverslipping, and imaging. To identify apoptotic cells, the Click-iT Plus TUNEL Assay Kit (C10618, Thermo Fisher Scientific) was used per the manufacturer’s instructions. The positive control included a slide treated with DNaseI (M6101, Promega) and the negative control excluded the Tdt enzyme. To colocalise apoptosis to neurons, slides were incubated in 10 mM citrate buffer in a steamer for 30 minutes. Slides were then incubated with NeuN-AF647 (1:100; ab190565, Abcam) with DAPI (1:1,000) for 30 minutes in the dark before washing, coverslipping, and imaging.

Slides were scanned using a ZEISS Axio Scan.Z1 (Zeiss) with a Plan-Apochromat 20×/0.45 M27 objective and acquired using ZEISS Zen slidescan (Sydney Microscopy and Microanalysis, The University of Sydney) or imaged on a Hamamatsu S360 scanner at 20 × resolution.

### T cell, microglia, PCNA, TUNEL, and MBP quantification.

Images of CD3-stained sections were analyzed using Visiopharm (Hørsholm, Denmark). T cell counts were normalized to brain area. Quantification of microglia density (number of microglia per mm^2^) in the cortex was performed manually using NDP.view2 software (Hamamatsu). First, the area of the cortex from sagittal sections was determined by manually drawing regions of interest followed by counting the number of microglia. Images of PCNA, TUNEL, and MBP-stained sections were analyzed with QuPath (59). Positive cell detection was used to obtain the percentage of PCNA-positive cells based on DAPI staining. TUNEL-positive cells were limited to colocalized match of TUNEL and DAPI staining. These were classified as TUNEL-positive cells and if they further colocalized with NeuN, were classified as apoptotic neurons; otherwise, they were classified as nonneuronal apoptotic cells. The number of apoptotic cells was normalized to area. In the cerebellum, MBP was most prominent in the white matter. White matter was isolated from the granule cell layer, molecular layer, and deep cerebellar nuclei using a trained pixel classifier, and MPB was quantified by the DAB staining intensity feature.

### DGE-Seq and bioinformatics.

Next generation sequencing libraries were generated using QuantSeq 3′ mRNA-Seq Library Prep FWD Kit (015.96, Lexogen) according to the manufacturer’s instructions. Libraries were sequenced as 75 bp fragments with a median depth of 4.2 million reads per sample on an Illumina NextSeq500. Gene quantitation was performed with Salmon (v 0.7.2) and Ensembl build 81. Generated counts were analyzed using edgeR (v 3.34) (60). Samples with a library size below 500,000 reads were excluded. Significance was considered with a FDR *P*_adj_ < 0.05. IFN score was calculated based on median of the fold changes for *Ifi27*, *Ifi44l*, *Ifit1*, *Isg15*, *Rsad2,* and *Siglec1* (8) for each genotype and treatment compared with vehicle-treated WT samples. Pathway analysis of significant genes was performed using QIAGEN IPA (QIAGEN Inc., https://digitalinsights.qiagen.com/IPA) (61).

### NF-H assay.

Neurofilament was measured on Protein Simple Ella using the NF-H Simple Plex Cartridge (SPCKB-PS-000519, Protein Simple). Serum samples were diluted 1:2 using the provided diluent and 50 μl of each diluted sample was loaded onto cartridge wells.

### Statistics.

Statistical analyzes were performed using GraphPad Prism (v8) (GraphPad Software) or edgeR and includes 1-way ANOVA, 2-way ANOVA, Kruskal-Wallis test, or quasi-likelihood F-test with specific statistical tests given in figure legends. Normality of residuals and homogeneity of variance assumptions were examined with Q-Q and homoscedasticity plots, respectively. Outliers were removed based on these graphs and the ROUT method. Data from qPCR, T cell counts, microglia density, PCNA, TUNEL, and MBP quantifications, and NF-H underwent log, squared, or square-root transformations to attain a normal distribution of residuals and homogeneity of variance, and, if these assumptions were not met, nonparametric tests were used. Data were presented as mean ± SEM or otherwise specified in the legend. *P* < 0.05 was considered significant.

### Study approval.

All studies were approved by the institutional animal care and use committee at Ionis Pharmaceuticals and were conducted in accordance with the US Public Health Service’s Policy on Humane Care and Use of Laboratory Animals. Some breeding of mice was done at the University of Sydney and was performed in compliance with the NSW Animal Research Act and its associated regulations and the 2013 NHMRC ‘Australian code of practice for the care and use of animals for scientific purposes’. Ethical approval was granted by the University of Sydney Animal Care and Ethics Committee (2014/688).

### Data availability.

The accession number for the DGE-Seq data is GEO: GSE231627. All supporting data are provided in the Supporting Data Values file or will be provided by the corresponding author upon request.

## Author contributions

BV, CH, FK, and MJH designed the study. BV, CH, CM, KH, and FK conducted the experiments, acquired data, and analyzed results. ILC developed the GIFN mice and contributed to critical discussion of results. TCF contributed to planning and data analysis and SD assisted in gene expression analysis. NBG provided histological analysis support and performed cortical microglia density analysis. BV and MJH wrote the manuscript. All authors reviewed and edited the manuscript.

## Supplementary Material

Supplemental data

Supporting data values

## Version 1 02/15/2024

Electronic publication
