# Supplementary material for: Interferon-α receptor antisense oligonucleotides reduce neuroinflammation and neuropathology in a mouse model of cerebral interferonopathy
Source: J Clin Invest. 2024 Feb 15;134(4):e169562. doi: 10.1172/JCI169562 (PMC10869178; doi:10.1172/JCI169562)
Supplement: Supplemental data [file jci-134-169562-s018.pdf]

Supplemental material to "Interferon-α receptor antisense oligonucleotides reduce neuroinflammation and neuropathology in a mouse model of cerebral interferonopathy"

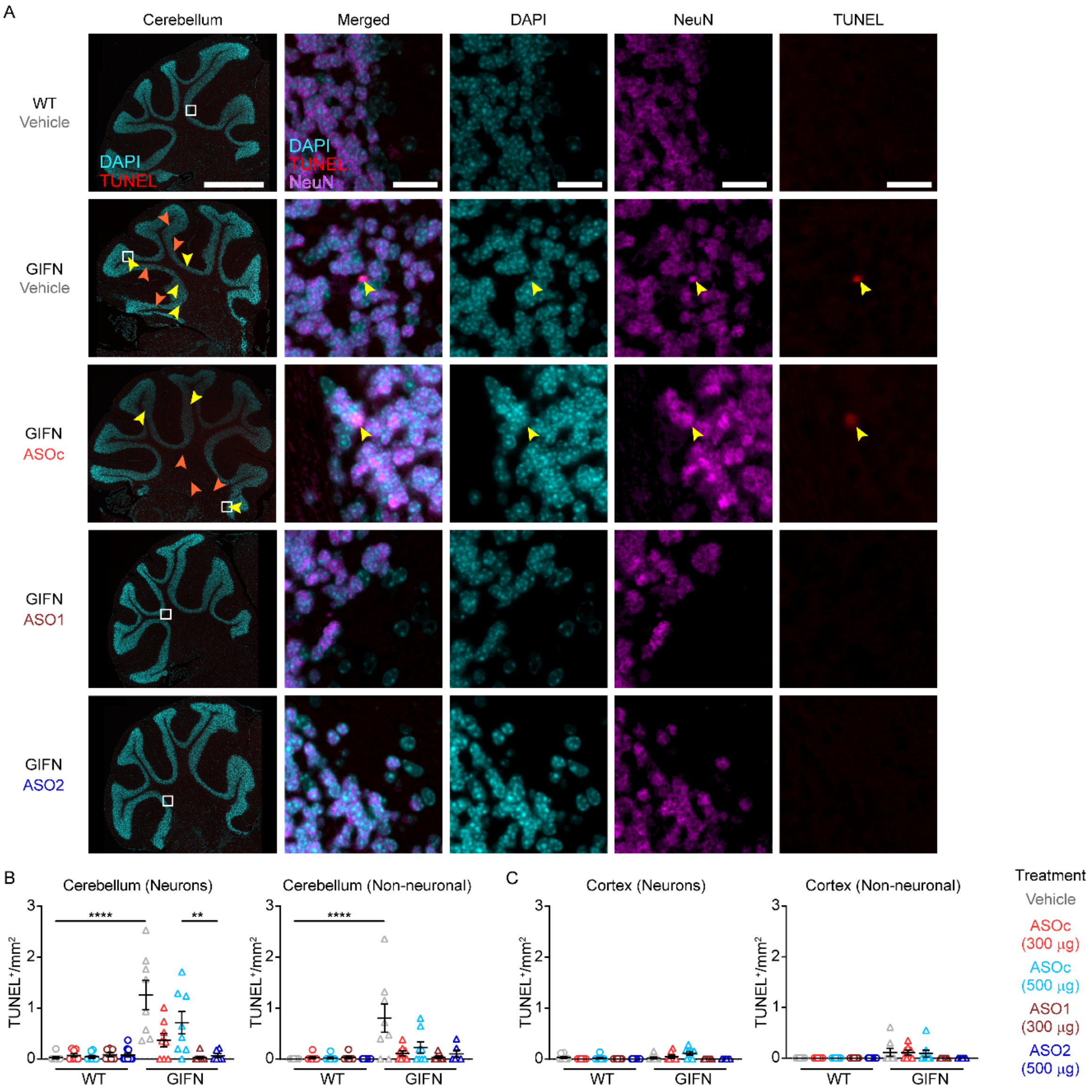

Supplemental Figure 1. Reduced apoptosis of cells in the brains of GIFN mice with ASO treatment. (**A**) Immunofluorescence for apoptotic cells (TUNEL-positive) and neurons (NeuN-positive) in the brains of WT and GIFN mice with different treatments (*n* = 6-10 per genotype per treatment). Representative images of the cerebellum are shown. White boxes indicate the region of the enlarged images. Yellow arrowheads point to colocalized TUNEL, NeuN and DAPI staining indicating an apoptotic neuron and orange arrowheads point to colocalized TUNEL and DAPI staining indicating an apoptotic non-neuronal cell. Scale bar: 1000 µm for cerebellum,

20 μm for enlarged images. Quantification of the number of apoptotic neurons and non-neuronal cells in the (**B**) cerebellum and (**C**) cortex. Each point is a mouse and mean and SEM are shown. $^{**}P < 0.01$, $^{***}P < 0.001$, by two-way ANOVA with Tukey post-test.

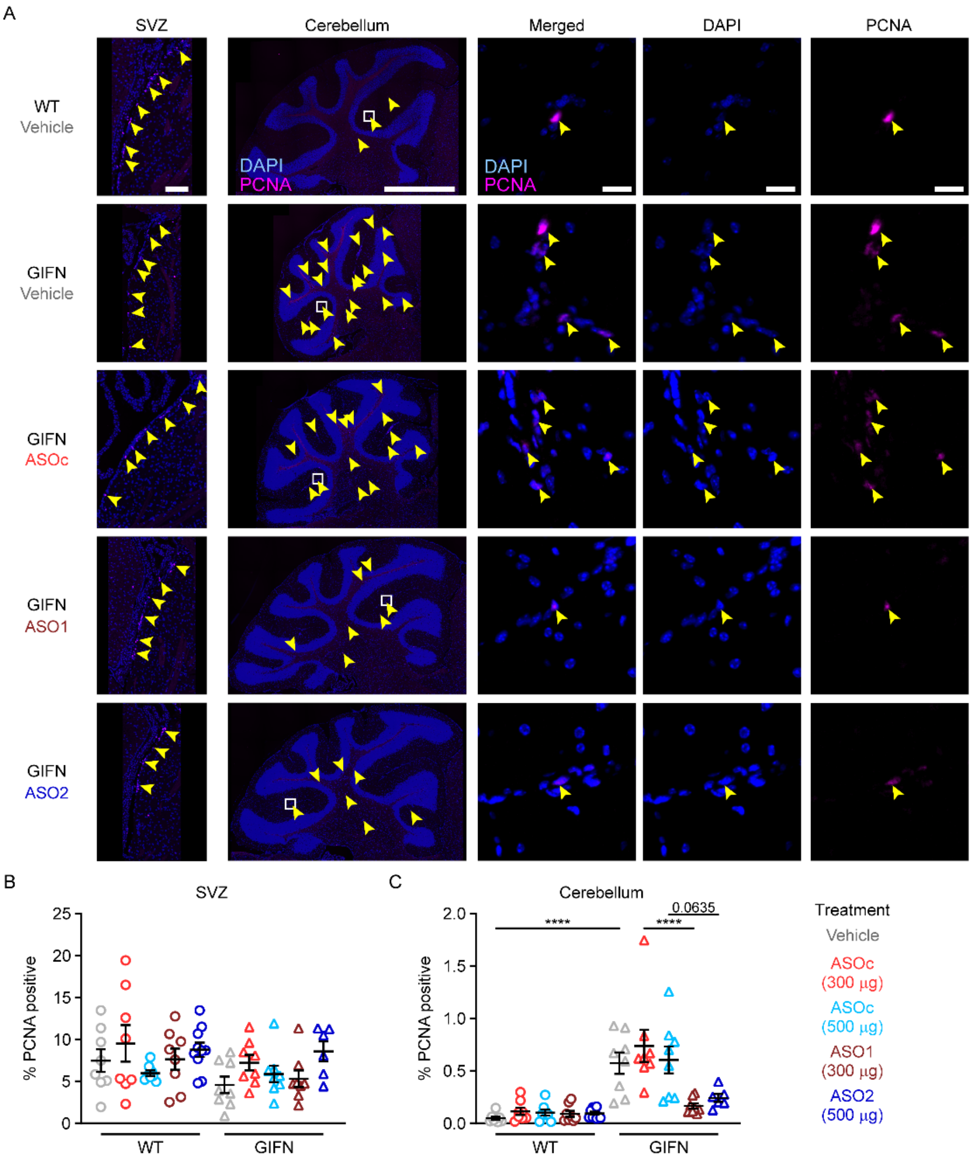

Supplemental Figure 2. Mouse *Ifnar1* ASOs partially reverse the number of proliferating cells in the cerebellum of GIFN mice. (**A**) Immunofluorescence for proliferating cells in the brain of WT and GIFN mice with various treatments (*n* = 6-10 per genotype per treatment). Representative images of the subventricular zone (SVZ) and the cerebellum are shown. White boxes indicate the region of the enlarged images (center three panels). Yellow arrowheads point to colocalized PCNA and DAPI staining indicating a proliferating cell. Scale bar: 1000 μm for cerebellum, 20 μm for enlarged images and 100 μm for SVZ. Quantification of the number of PCNA-positive cells relative to the number of cells in (**B**) SVZ and (**C**) cerebellum. Each point is a mouse and mean and SEM are shown. ****$P < 0.0001$, by two-way ANOVA with Tukey post-test.

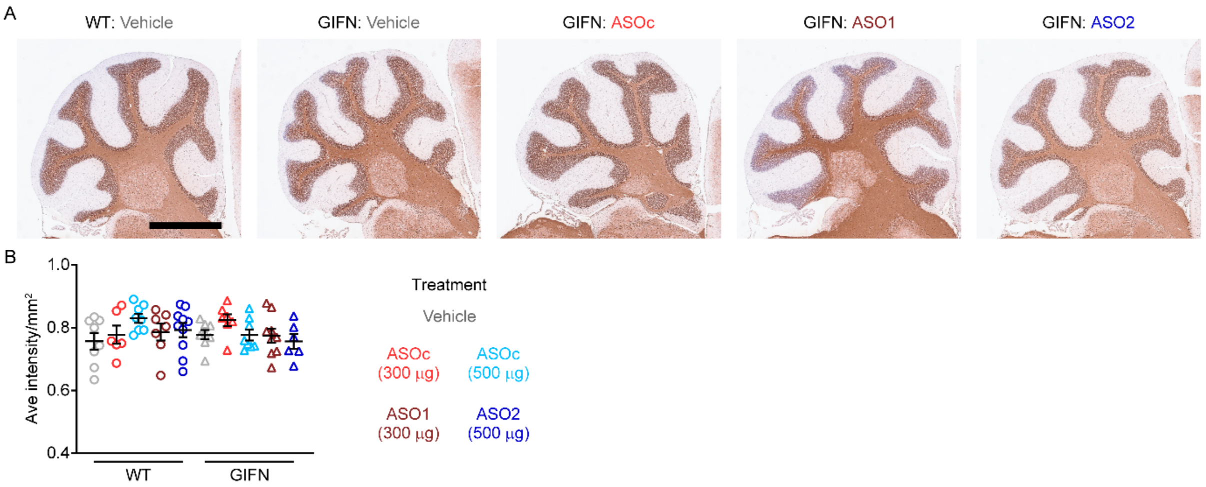

Supplemental Figure 3. No detectable change in myelin staining between WT and GIFN mice. (**A**) Representative images of the cerebellum stained for MBP (brown) and counterstained for nuclei (purple). There were no observable differences in the staining pattern in the white matter of all mice (n = 6-10 per genotype per treatment). Scale bar: 1000 µm. (**B**) Quantification of the average staining intensity per mm^2 of the white matter in the cerebellum. Each point is a mouse and mean and SEM are shown. No significant differences measured by two-way ANOVA with Tukey post-test.

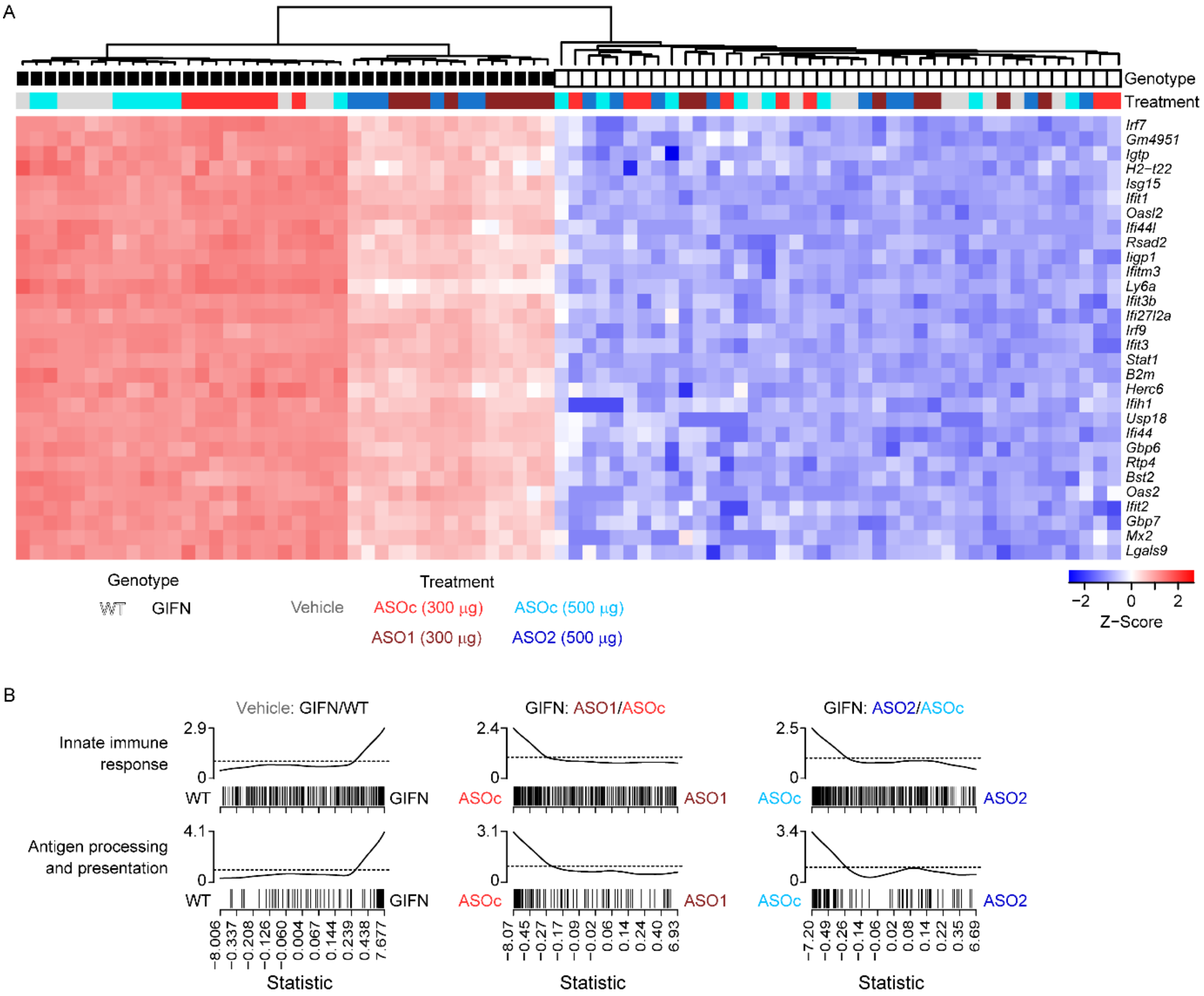

Supplemental Figure 4. Mouse *Ifnar1* ASOs partially reverse expression changes of regulated genes in GIFN mice compared with WT mice. (**A**) Heatmap of gene expression of the top genes differentially regulated in GIFN vs WT vehicle-treated mice across all samples. (**B**) Barcode plots of ontology terms indicate the enrichment of genes associated with the term with the plot (solid line) depicting the relative enrichment.

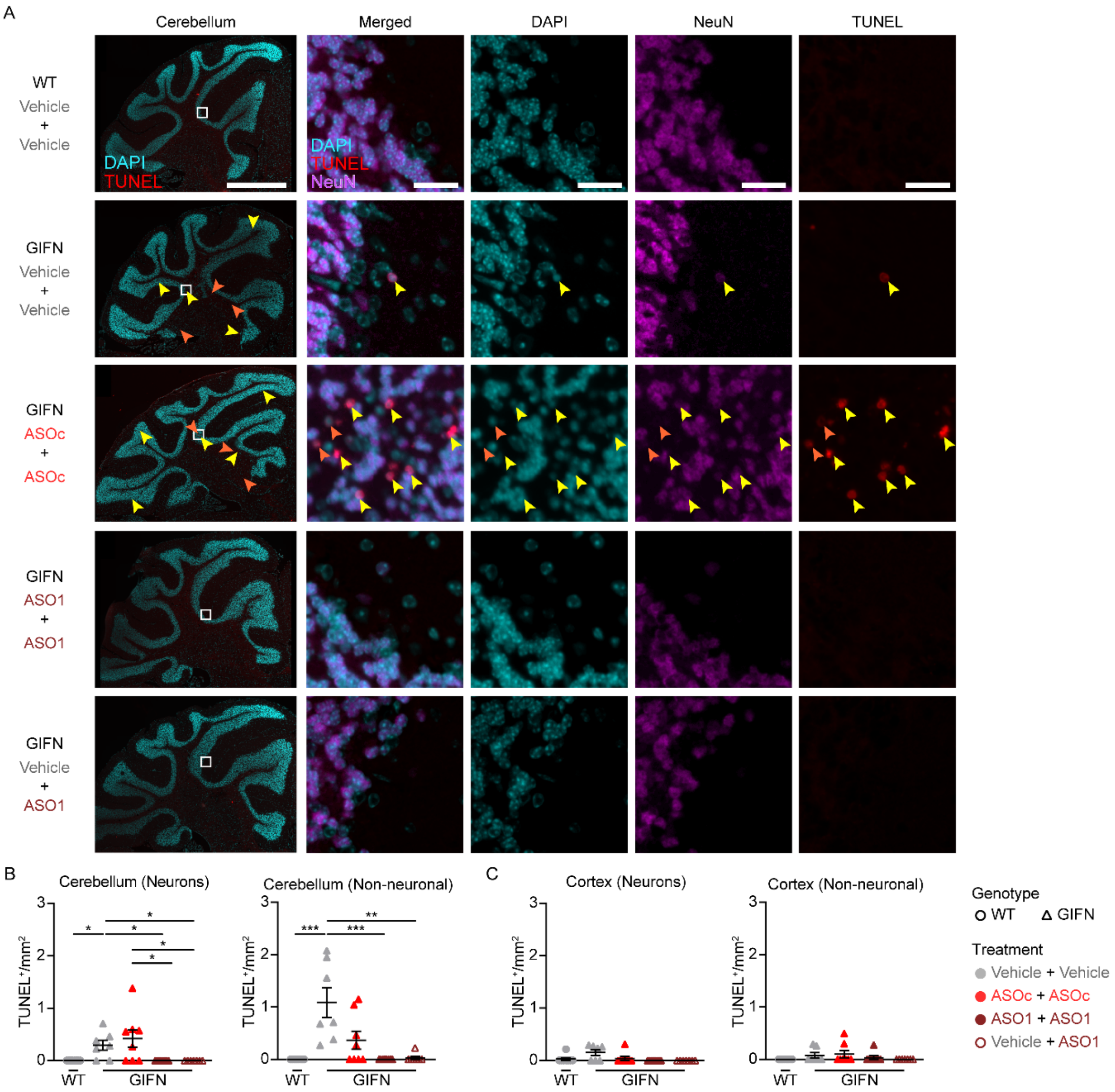

Supplemental Figure 5. Reduced apoptosis of cells in the brains of GIFN mice with delayed ASO treatment. (**A**) Immunofluorescence for apoptotic cells (TUNEL-positive) and neurons (NeuN-positive) in the brains of WT and GIFN mice with different treatments (*n* = 6-10 per genotype per treatment). Representative images of the cerebellum are shown. White boxes indicate the region of the enlarged images. Yellow arrowheads point to colocalized TUNEL, NeuN and DAPI staining indicating an apoptotic neuron and orange arrowheads point to colocalized TUNEL and DAPI staining indicating an apoptotic non-neuronal cell. Scale bar: 1000 μm for cerebellum, 20 μm for enlarged images. Quantification of the number of apoptotic neurons and non-neuron cells in the (**B**) cerebellum and (**C**) cortex. Each point is a mouse and mean and SEM are shown. *$P < 0.05$, **$P < 0.01$, ***$P < 0.001$, by Kruskal-Wallis test with Dunn's post-test.

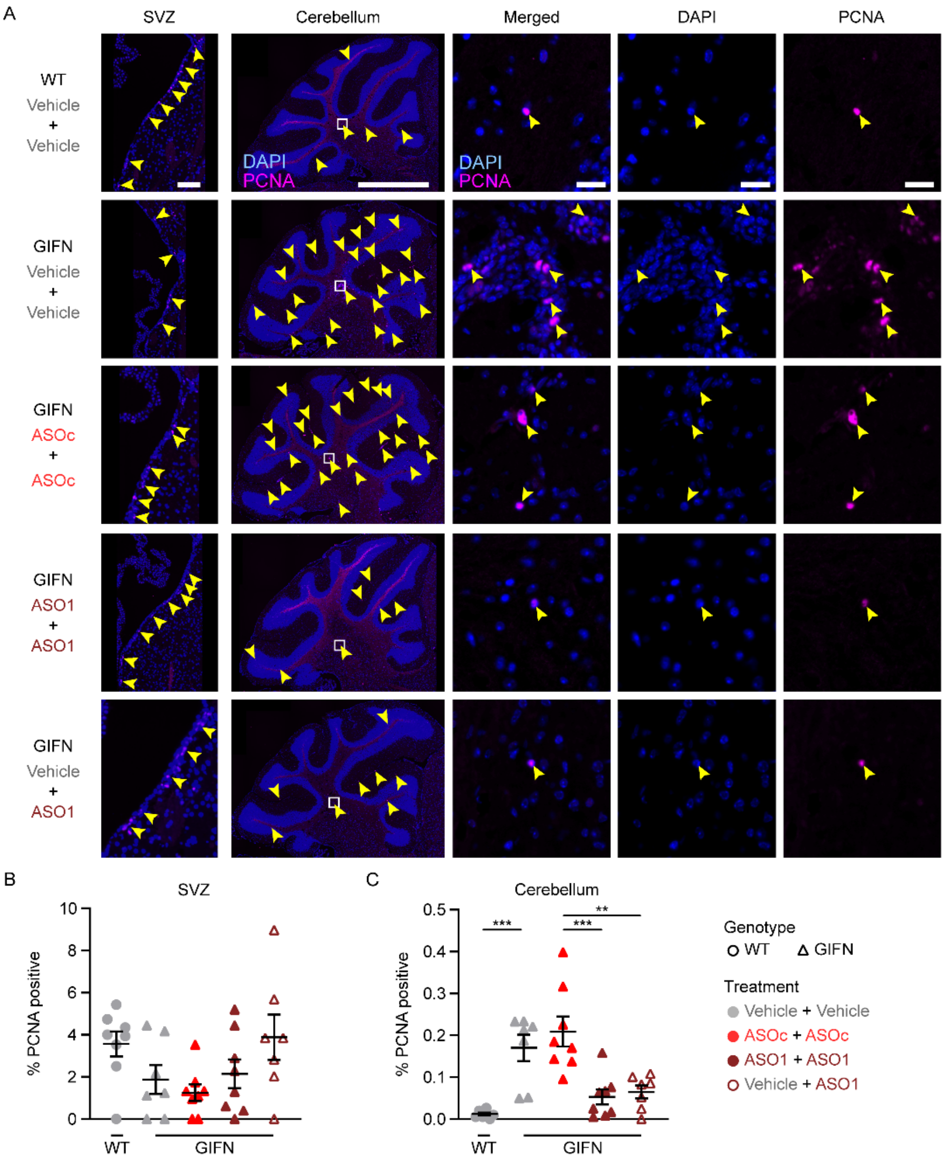

Supplemental Figure 6. Delayed ASO treatment partially reverses the number of proliferating cells in the cerebellum of GIFN mice. (**A**) Immunofluorescence for proliferating cells in the brain of WT and GIFN mice with various treatments (*n* = 6-10 per genotype per treatment). Representative images of the subventricular zone (SVZ) and cerebellum are shown. White boxes indicate the region of the enlarged images (center three panels). Yellow arrowheads point to colocalized PCNA and DAPI staining indicating a proliferating cell. Scale bar: 1000 μm for cerebellum, 20 μm for enlarged images and 100 μm for SVZ. Quantification of the number of PCNA-positive cells relative to the number of cells in (**B**) SVZ and (**C**) cerebellum. Each point is a mouse and mean and SEM are shown. $^{**}P < 0.01$, $^{***}P < 0.001$, by one-way ANOVA with Tukey post-test.
